# Effect of amitriptyline on orthodontic tooth movement in rats: an experimental study

**DOI:** 10.34172/joddd.2020.033

**Published:** 2020-09-21

**Authors:** Mohammad Sadegh Ahmad Akhoundi, Mahdiyeh Shaygan-Mehr, Mohammad Ali Keshvad, Shahroo Etemad Moghaddam, Mojgan Alaeddini, Ahmadreza Dehpour, Amir Hossein Mirhashemi

**Affiliations:** ^1^Dental Research Center, Dentistry Research Institute, Tehran University of Medical Sciences, Tehran, Iran; ^2^DDS, Tehran University of Medical Sciences, Tehran, Iran; ^3^Department of Orthodontics, School of Dentistry, Tehran University of Medical Sciences, Tehran, Iran; ^4^Department of Pharmacology, Faculty of Medicine, Tehran University of Medical Sciences, Tehran, Iran

**Keywords:** Amitriptyline, Bone density, Rats, Tooth movement techniques

## Abstract

**Background.** Orthodontic tooth movement (OTM) occurs in the alveolar bone; therefore, any condition affecting bone quality can alter OTM. This study aimed to evaluate the effect of amitriptyline on OTM in rats.

**Methods.** Forty-five male Wistar rats were randomly divided into three groups: (I) no injection, (II) injection with saline solution, and (III) injection of amitriptyline. Next, a 60-gr force was applied to the maxillary left first molar tooth of all the rats, using a nickel‒titanium closed-coil spring ligated between the maxillary incisors and the left first molar tooth. The rats were sacrificed after 21 days to measure OTM and perform histological analysis to determine the number, width, and depth of resorptive lacunae, osteoclast counts, and periodontal ligament (PDL) width.

**Results.** The highest and the lowest OTM rates were found in the control and amitriptyline groups, respectively; however, there was no significant difference between the study groups in this regard. Histological analysis showed a significantly lower number of resorption lacunae in the amitriptyline group than the saline group.

**Conclusion.** Although no significant difference was noted in OTM after amitriptyline administration, a reduction in the number of resorptive lacunae in rats injected with amitriptyline suggests that amitriptyline affects the bone tissue at the cellular level.

## Introduction

Researchers are searching for strategies to enhance the rate of orthodontic tooth movement (OTM) to improve orthodontic treatment efficacy and provide better anchorage control. A tooth’s position in the dental arch can be changed by orthodontic treatment via remodeling of the surrounding bone. This process is modulated by osteoblasts that are mainly responsible for bone formation, and osteoclasts which are responsible for bone resorption by secretion of proteolytic enzymes and acids.^1^

OTM is an inflammatory process that depends on the presence of inflammatory mediators, i.e., cyclic nucleotides,^2^ neurotransmitters,^3^ and prostaglandins.^4^ Recent studies have emphasized the role of medications such as non-steroidal anti-inflammatory drugs (NSAIDs), nitric oxide, and leukotriene inhibitors on OTM. It has been suggested that high doses of NSAIDs, bisphosphonate, and leukotriene inhibitors can decrease OTM due to their anti-inflammatory effects.^5^

Unipolar depression is the most prevalent mental disorder worldwide, and its key hallmarks include sadness, decreased self-esteem, and indifference towards everyday activities.^6^ Tricyclic antidepressants (TCAs) are the primary medications prescribed for depression since the 1980s.^7^ Amitriptyline, amoxapine, trimipramine, nortriptyline, and imipramine are the most common TCAs available on the market.^8^ A previous study reported a complicated relationship between depression, drug intake, and bone fracture. The hypothesis was that depression might increase the chance of falling, and at the same time, a reduction in bone density induced by medications could increase the possibility of fracture.^9^ In other words, elderly patients taking antidepressants are at higher risk of bone fracture; however, this association has not been clearly proven. Precise evaluation of the effects of antidepressants on bone density is difficult because depression itself has an impact on the neurotransmitter balance and bone density.^10,11^ As stated by Haney et al,^12^ the results of studies on the association of bone mineral density, fracture risk, and intake of antidepressant are negatively affected by the confounding factors because depression has a possible association with both bone mineral density/fracture and medications taken.

Amitriptyline is one of the most commonly prescribed TCAs for managing mental conditions, neuropathic pains, and fibromyalgia. It suppresses the production and secretion of nitric oxide and prostaglandin E2 by 16‒27%, and acts as an anti-inflammatory agent, leading to inhibitory effects on the bone remodeling process.^13^ On the other hand, similar to selective serotonin reuptake inhibitors (SSRIs), amitriptyline is known to block the serotonin transporter gene (5-HTT), balance the transmission of noradrenaline, and inhibit serotonin and noradrenaline reuptake, which can cause bone resorption and osteoporosis as shown by Calarge et al.^2,14^ Therefore, amitriptyline might exert a dual effect on the rate of OTM. Considering the high prevalence of depression and lack of studies on the effects of TCAs on OTM and alveolar bone remodeling, this study aimed to evaluate the effect of amitriptyline on OTM in rats in a reasonable time.

## Methods

This study was conducted following the guidelines for the care and use of laboratory animals and was approved by the Ethics Committee of Tehran University of Medical Sciences. Forty-five male Wistar rats with an initial weight of 220‒250 g were obtained from the Department of Pharmacology, Tehran University of Medical Sciences, and randomly assigned to three groups (n=15) as follows:

**Saline (S):** The rats received daily intraperitoneal injections of 1 mL of saline solution.

**No injection:** The rats received no injection at all.

**Amitriptyline:** The rats received a daily intraperitoneal injection of 10 mg/kg amitriptyline.

The rats were kept in standard conditions in terms of temperature and lighting (24-hour light/24-hour dark cycles).

Amitriptyline was provided by Pars Darou Company (Tehran, Iran) and dissolved in a saline solution every day before injection under laboratory conditions. Its dosage was adjusted according to the rats’ weights. The amount of the injected medication was determined based on the doses prescribed in a similar previous study.^15^ Next, 1 mL of each solution was injected intraperitoneally using an insulin syringe every afternoon for 21 days.

**Figure 1:**
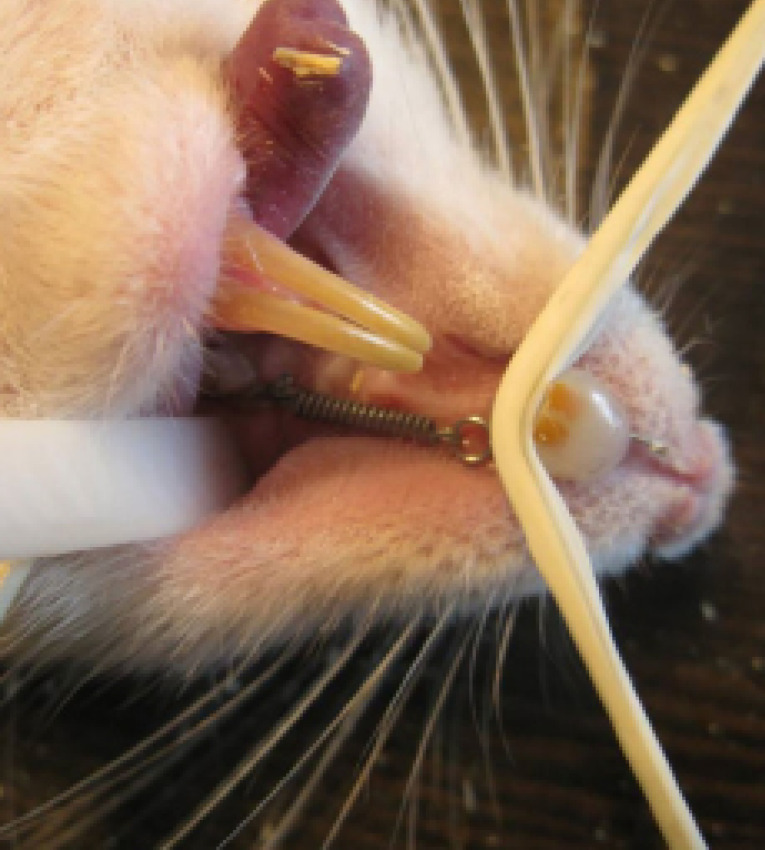
Appliance view after fixation.

After induction of anesthesia with a combination of 50 mg of ketamine and 6 mg of xylazine, shallow grooves (<1 mm) were created on the labial and distal surfaces of both incisors using a dental handpiece. Orthodontic appliances were designed and placed between the incisors and left molars to induce orthodontic movement of the left first molar. These consisted of 0.01-inch ligature wires attached to both ends of a NiTi closed-coil spring with an initial length of 6 mm. Distally, the ligatures passed through the contact area of the first and second molars and were secured at the cervical area of the first molar. Mesially, they were placed between the two incisors and situated in the previously prepared grooves, followed by etching with 37% phosphoric acid and application of 3M bonding agent and 3M Unitek Transbond^TM^ XT light-cured adhesive paste. To provide better anchorage and maximum retention for the ligature wires, the labial, distal, and palatal surfaces of both incisors were covered with composite resin (Figure 1). After appliance fixation, the incisal edges of the lower incisors were reduced by 2 mm to avoid ligature wire damage and appliance displacement by the teeth. The spring force was adjusted to apply an initial force of 60 g using a Dentaurum stress and tension gauge while ligating the wire around the incisors.

Due to the fast eruption rate of mandibular incisors in rats, the animals were anesthetized once a week, the appliance position was checked, and the incisal edges were reduced as required. To decrease the probability of appliance breakage or displacement, food was delivered in powdered form to the rats during the study.

After 21 days of force delivery, all the rats were sacrificed, their maxillae were resected, and the distance between the first and second molars was measured by a feeler gauge.

### Histological analysis

After recording the required measurements, the appliances were removed, and the maxillae were fixed in 10% formalin for 48 hours, followed by immersion in 5% formic acid until decalcification was complete. Routine histological processing was carried out, and 4-µm parasagittal sections of the molars were cut, followed by hematoxylin-and-eosin (HE) staining. Three slides of each sample, including the largest root surface, were selected and the mesial root of the first molar was analyzed by two blinded oral and maxillofacial pathologists under a light microscope (BX51; Olympus, Tokyo, Japan) equipped with a digital camera (DP25; Olympus, Tokyo, Japan) and a software program (DP2-BSW; Olympus, Tokyo, Japan). The mean values of these sections were recorded as the final measurement for each animal, and disagreements between the observers were resolved by discussion until a consensus was reached.

**Figure 2:**
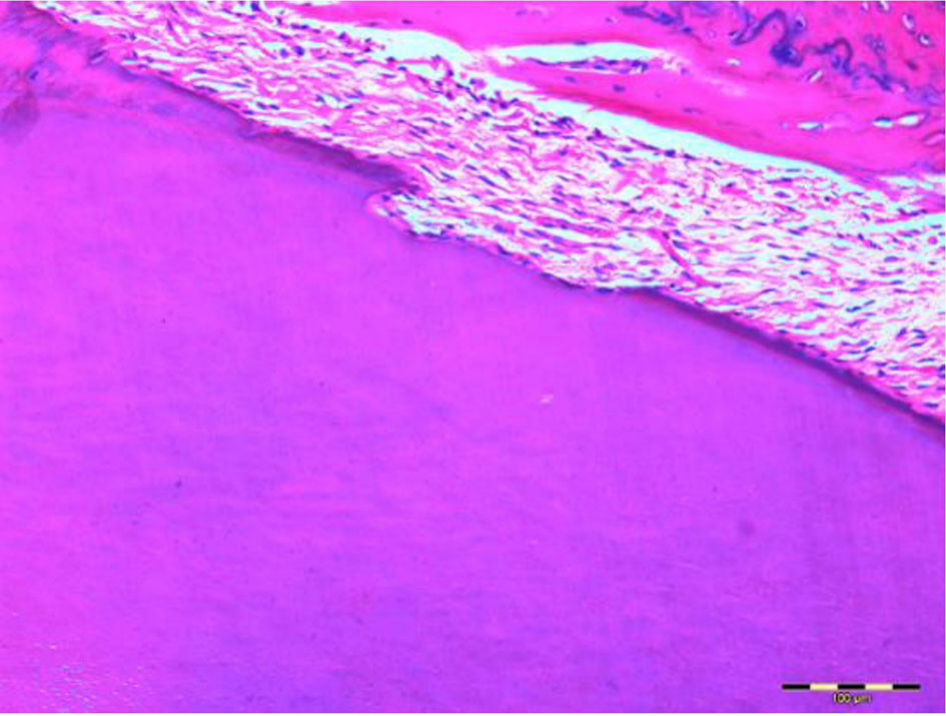
Microscopic section of the mesial root of the first molar and alveolar bone containing resorptive lacuna (magnification ×400).

**Figure 3:**
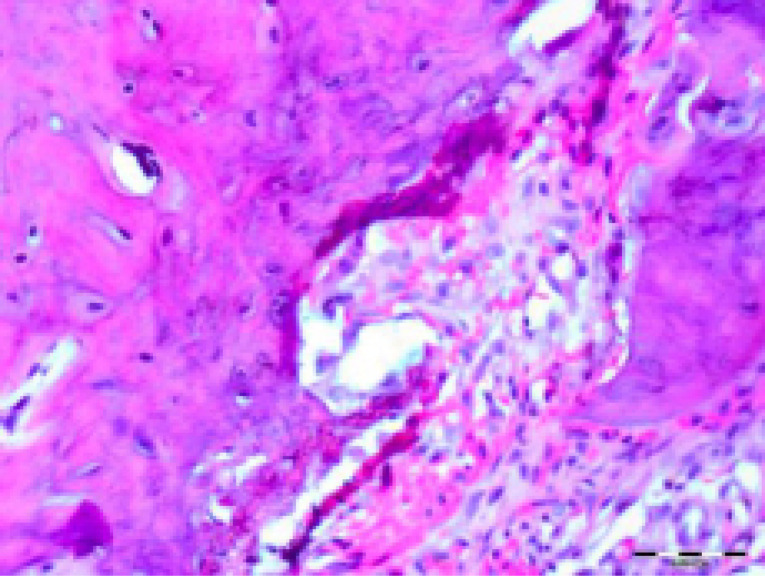
Microscopic section of the mesial root of the first molar and alveolar bone containing osteoclasts (magnification ×400).

The width and depth of the resorptive lacunae (Figure 2) were assessed on both the mesial and distal surfaces of the mesial root of the first molars, in addition to counting the osteoclasts, to determine root resorption.^16^ As demonstrated in Figure 3, osteoclasts were identified as multi-nucleated cells with abundant, slightly basophilic to bright, eosinophilic cytoplasm.

Periodontal ligament (PDL) width was calculated as the space between the root surface and the alveolar bone in the most coronal and apical parts of both the mesial and distal root surfaces.^17^

### Statistical analysis

One-way ANOVA was used for pairwise comparisons. Considering the homogeneity of variances, the Tukey’s HSD test was also applied. The Kruskal-Wallis test was carried out, as well. P<0.05 were considered significant.

## Results

### Tooth movement

All the groups exhibited some degrees of OTM on the appliance side after 21 days, as depicted in Table 1. The mean OTM was the highest in the control group, followed by the saline solution group, with the lowest in the amitriptyline group; however, one-way ANOVA revealed no significant difference between the study groups (P>0.05).

**Table 1:** Descriptive data regarding tooth movement in the study groups

**Groups**	**Mean (mm)**	**SD**	**Maximum**	**Minimum**
**Amitriptyline**	0.28	0.24	0.75	0.03
**Control**	0.37	0.27	1.00	0.10
**Saline**	0.33	0.19	0.80	0.15

### Histological analysis

Statistical analysis showed no significant differences in osteoclast counts, neither in the mesial nor in the distal surface (Table 2; P>0.05). There was no significant difference in the depth or width of the resorptive lacunae between the study groups (P>0.05) as presented in Tables 3 and 4; however, there was a significant difference in the number of lacunae between the saline solution (mean=2.21) and amitriptyline (mean=1.69) groups at the mesial surface of the examined root (Table 5; P=0.038).

**Table 2:** Osteoclast counts at the mesial and distal surfaces of the mesial root of the maxillary first molar (P=0.855 and P=0.409, respectively)

**Groups**	**Mean**		**SD**		**Maximum**		**Minimum**	
	**Mesial**	**Distal**	**Mesial**	**Distal**	**Mesial**	**Distal**	**Mesial**	**Distal**
**Amitriptyline**	0.77	1.00	0.83	100	3	2	0.0	0.0
**Control**	0.93	0.71	1.38	1.48	5	5	0.0	0.0
**Saline**	1.21	0.64	1.52	0.92	5	3	0.0	0.0

**Table 3:** The width of resorptive lacunae at the mesial and distal surfaces of the mesial root of the maxillary first molar (P=0.364 and P=0.308, respectively)

**Groups**	**Mean (µm)**		**SD**		**Maximum**		**Minimum**	
	**Mesial**	**Distal**	**Mesial**	**Distal**	**Mesial**	**Distal**	**Mesial**	**Distal**
**Amitriptyline**	182.32	109.08	155.54	100.06	565.10	249.03	0.0	0.0
**Control**	210.74	217.68	128.52	272.49	381.50	813.90	0.0	0.0
**Saline**	283.20	233.98	169.54	210.63	640.00	763.10	0.0	0.0

**Table 4:** The depth of resorptive lacunae at the mesial and distal surfaces of the mesial root of the maxillary first molar (P=0.425 and P=0.285, respectively)

**Groups**	**Mean (µm)**		**SD**		**Maximum**		**Minimum**	
	**Mesial**	**Distal**	**Mesial**	**Distal**	**Mesial**	**Distal**	**Mesial**	**Distal**
**Amitriptyline**	33.99	28.58	22.34	28.63	71.80	92.60	0.0	0.0
**Control**	38.19	39.55	25.69	40.78	88.50	104.5	0.0	0.0
**Saline**	52.98	48.02	39.68	29.65	137.7	104.9	0.0	0.0

**Table 5:** Resorptive lacunae counts at the mesial and distal surfaces of the mesial root of the maxillary first molar (P=0.038 and P=0.145, respectively)

**Groups**	**Mean**		**SD**		**Maximum**		**Minimum**	
	**Mesial**	**Distal**	**Mesial**	**Distal**	**Mesial**	**Distal**	**Mesial**	**Distal**
**Amitriptyline**	1.69	1.00	1.03	1.00	3	2	0.0	0.0
**Control**	2.36	0.71	1.59	1.48	6	5	0.0	0.0
**Saline**	2.21	3.07	1.42	1.49	4	5	0.0	0.0

The PDL width was measured at four different root surfaces of the mesiobuccal root (mesioapical, distoapical, mesiocoronal, and distocoronal surfaces), and was not significantly different in either of the measurement points among the groups (P>0.05).

## Discussion

Numerous studies have evaluated the localized and systemic effects of medications and nutritional supplements on OTM. Thus, clinicians should have adequate knowledge in this respect due to the high chance of intake of such medications by orthodontic patients.^16,18,19^

In order to evaluate the effects of such medications on bone remodeling, in vitro studies might not accurately reflect the exact conditions in living organisms, due to their limitations in simulating the precise biochemical and biomechanical processes that occur in vivo. Despite biological differences between animals and humans, animal models are preferred to experiments on cell cultures. We chose rats for this study because of their easy reproduction and maintenance, compared with dogs or cats.

Force application to molar teeth from the mesial direction is among the most common designs adopted by similar investigations; therefore, we fabricated an appliance to induce this type of movement. Since NiTi enables the application of continuous light force during the test period, we used NiTi closed-coil springs in the present study.^20^ As the bone remodeling cycle in rats takes 14 days according to similar studies^21,22^ and in order to assess the long-term effects and stabilization of the respective medication, the duration of force application and medication intake was 21 days. Amitriptyline was injected intraperitoneally at a dose of 10 mg/kg, similar to a previous study.^15^

Mental stresses probably affect the hypothalamus-hypophysis axis and the immune system and induce changes in osteoclastic activity. Antidepressants and benzodiazepines that are routinely prescribed to treat these conditions can have different side effects on patients. Increased root resorption during orthodontic treatment of patients suffering from such disorders has been previously reported.^23^

TCAs such as amitriptyline affect the serotonin-noradrenaline system and are commonly prescribed for >10% of the US population for the treatment of depression.^24^ These medications can inhibit inflammatory responses, and at the same time, decrease bone density, resulting in a higher rate of bone fractures similar to SSRIs.^25^ Therefore, it appears that amitriptyline might have a dual effect on OTM. A recent meta-analysis on the effects of antidepressants, including TCAs and SSRIs, on the bone mineral density of women, showed that this association has not been studied in detail, and only four studies fulfilled the authors’ inclusion criteria. They concluded that there seems to be no relationship between the use of TCAs and bone mineral density.^26^

The present study evaluated the effect of amitriptyline on OTM in rats. This medication was selected due to its mechanism of action, which is similar to that of SSRIs and also its anti-inflammatory properties.

Rizzoli et al^9^ reported that amitriptyline, as a depression medication, can increase the risk of bone fractures. Based on this finding, it was hypothesized that amitriptyline administration might induce bone density reduction and accelerate bone resorption following the application of orthodontic forces; however, the present study did not confirm this hypothesis. The amitriptyline group was not significantly different from the other two groups in the rate of OTM or drug-related bone resorptive factors. Although the numerical value of OTM decreased in rats receiving amitriptyline, a definite conclusion regarding the effects of this medication on bone metabolism cannot be drawn due to the insignificance of the findings in this study.

Yaron et al^27^ reported that fluoxetine and amitriptyline could decrease NO release in a dose-dependent manner and indirectly block prostaglandin E2 secretion. Accordingly, we expected to find a significantly diminished OTM rate; however, despite the lower mean rate of OTM in the amitriptyline group, it did not reach statistical significance and therefore, our findings could not clarify the anti-inflammatory effects of amitriptyline, keeping in mind that we did not determine the level of inflammatory factors.

Petronijevic et al^6^ evaluated premenopausal women with mono-polar depression in terms of the duration of depression, medical (antidepressive) treatment, and hormonal status. They reported that patients suffering from this condition had a decreased bone density, irrespective of the type of medication (SSRIs, TCAs, etc.) they were taking, and the amount of bone density reduction had a direct association only with the period and severity of depression, not with hormonal changes or medications prescribed. Bone density was not evaluated in the current study; however, since no difference was found in OTM between the study groups, it seems that these findings might somehow confirm their results in that the effect of TCAs on bone density is minimal if any.

In the present study, there was no significant difference in root resorption between the groups, except for the resorptive lacuna count between the saline solution and amitriptyline groups. Lahoti et al^23^ reported that osteoclastic activity changed in depressed patients, and root resorption was more prevalent in these individuals after force application. However, it was not clarified whether this was related to the disorder itself or a side effect of medication. According to the results of the current study, the number of lacunae was significantly lower in the amitriptyline group than the saline solution group. However, none of the other resorption-related variables, such as osteoclast counts or resorptive lacunae width/depth, were significantly different between the study groups. We cannot conclude that the significantly lower number of lacunae in the amitriptyline group was drug-related, especially considering that osteoclast counts and activity are not always positively correlated.^18^ In a similar previous study, Mirhashemi et al^28^ investigated the association of fluoxetine consumption with OTM in rats and concluded that tooth movement increased in the fluoxetine group, while bone density in the alveolar bone, hard palate, skull, and mandibular bone significantly decreased. Consistent with the present study, there was no difference either in the width/depth of resorptive lacunae or osteoclast counts in their study. A possible reason for increased OTM might be the adverse effect of SSRIs on bone density, as mentioned by Tsapakis et al,^29^ in their review article in 2012,but this effect was not substantiated for amitriptyline in the present study.

One major limitation of this study was the similar timing of the administration of amitriptyline and onset of OTM. As we know, amitriptyline is taken orally by patients in clinical situations, and injection of antidepressants before the onset of OTM can be considered in future studies.

## Conclusion

Due to the limitations of this study, we cannot claim with certainty that amitriptyline affects the rate or quality of OTM, and more research is required to obtain conclusive evidence. Although the rate of OTM, osteoclast counts, width and depth of resorptive lacunae, and PDL width showed no significant difference between the study groups, a significant reduction in the resorptive lacunae counts in rats receiving amitriptyline was observed in this study. This finding, along with the previously reported effects of this medication on orthodontic patients, suggests that orthodontic treatment should be performed with caution in patients taking TCAs until further clarification is provided regarding the osseous side effects of these medications.

This research was carried out in the Dental Research Center, Dentistry Research Institute of Tehran University of Medical Sciences with the code number: 6021.

## Author Contributions

Conceptualization: MSAA. Data collection: AHM. Formal analysis: MAK. Investigation: SEM and MA. Methodology: MA. Project administration: MSAA. Resources: MSM. Software: MSM. Supervision: AHM. Validation: ARD. Visualization: ARD. Writing the original draft: MAK. Review and editing: SEM. All the authors have read and agreed to the published version of the manuscript.

## Acknowledgment

We surely want to acknowledge outstanding contribution of Dr. Mojdeh Kalantar Motamedi for English editing of this article.

## Funding

Not applicable.

## Competing Interests

The authors declare no competing interests with regards to the authorship and/or publication of this article. This research did not receive any specific grant from funding agencies in the public, commercial, or not-for-profit sectors.

## Ethical Approval

This study was conducted in accordance with the guidelines for the care and use of laboratory animals and was approved by the Ethic Committee of Tehran University of Medical Sciences. Code No. 6021.
