# Effect of amitriptyline on orthodontic tooth movement in rats: An experimental study

**DOI:** 10.34172/joddd.2023.40445

**Published:** 2023-07-17

**Authors:** Mohammad Sadegh Ahmad Akhoundi, Mahdiyeh Shaygan-Mehr, Mohammad Ali Keshvad, Shahroo Etemad-Moghadam, Mojgan Alaeddini, Ahmadreza Dehpour, Amir Hossein Mirhashemi

**Affiliations:** ^1^Dental Research Center, Dentistry Research Institute, Tehran University of Medical Sciences, Tehran, Iran; ^2^DDS, Tehran University of Medical Sciences, Tehran, Iran; ^3^Department of Orthodontics, School of Dentistry, Tehran University of Medical Sciences, Tehran, Iran; ^4^Department of Pharmacology, Faculty of Medicine, Tehran University of Medical Sciences, Tehran, Iran

In the article entitled "Effect of amitriptyline on orthodontic tooth movement in rats: an experimental study" which appeared in J Dent Res Dent Clin Dent Prospect 2020;14(3): 147- 152, doi: 10.34172/joddd.2020.033, the name of the fourth author has been misspelled. The correct spelling is Shahroo Etemad-Moghadam. The original version of the article has been updated to reflect the corrections.

